# Associations of Sedentary Behavior with Physical Fitness and Academic Performance among Chinese Students Aged 8–19 Years

**DOI:** 10.3390/ijerph16224494

**Published:** 2019-11-14

**Authors:** Xin Huang, Nan Zeng, Sunyue Ye

**Affiliations:** 1 Institute of Child Development, Jiaxing University, Jiaxing 314001, Zhejiang, China; huangxin3518@163.com; 2 Department of Food Science and Human Nutrition, Colorado State University, Fort Collins, CO 80523, USA; nanzeng@colostate.edu

**Keywords:** children, academic achievement, cardiorespiratory fitness, sedentary lifestyle, screen time

## Abstract

*Background*: Existing evidence regarding how time spent on various types of sedentary behavior (SB) in relation to physical fitness (PF) and academic performance (AP) in children and adolescents is limited. This cross-sectional study aimed to explore the associations of SB types with PF and AP among 8–19-year-old Chinese students. *Methods*: A total of 1164 students were recruited from five schools in Zhejiang province, China. Children’s SB was assessed by a valid questionnaire and AP was represented by scores on four courses. PF was measured by Chinese National Student PF Standard battery. The associations were assessed using linear mixed-effects models adjusted for age, school, grade, and class. *Results*: Students’ screen-based SB was observed to be negatively associated with PF in girls (*p* < 0.05). Screen-based SB was inversely associated with AP in both girls and boys (*p* < 0.001). The significant interaction between weekdays and weekends, and SB on AP was observed in girls (*p* < 0.001). On weekends, screen-based SB ≥ 6 h/day (<3 h/day as reference group) was significantly and negatively associated with AP (*p* < 0.01 for both sex). *Conclusions:* Longer screen-based SB, especially on weekends, was related to poorer AP in 8–19-year-old Chinese students. Our findings suggest that restrictions on any type of screen-based SB for leisure on weekends are beneficial to AP in Chinese students.

## 1. Introduction

Sedentary behavior (SB), any waking behavior characterized by an energy expenditure ≤1.5 METs (metabolic equivalent of tasks) while in a sitting or reclining posture, is a widespread lifestyle around the world for nowadays’ labor-saving society [1]. In China, nearly 70% of primary and high school students have not achieved the national recommendation of 150 min moderate-vigorous physical activity (MVPA) per day [2] and about 37% of them did not meet the recommendations for screen-based SB (i.e., no more than 2 h per day) [3,4]. SB was also an important risk factor for their immediate and delayed physical or mental health problems, including obesity, cardiovascular disease, self-esteem, social behavior disorder, and physical fitness in children and adolescents [5,6,7]. Moreover, Chinese students’ physical fitness has declined in recent years [8] and previous studies indicated that this physical weakness may be partially due to the insufficient MVPA and screen-based sedentary lifestyle [9,10]. Screen-based SB may have different effects on physical fitness for children compared with other types of SB because of its various sitting postures, energy expenditure, and time duration [11,12]. However, the current literature examining the relationships between SB types/components and physical fitness in Chinese students is limited.

Good academic performance/achievement is crucial for primary and middle school students in China where it is closely related to their entrance qualifications to universities and a successful beginning of their future careers [13,14]. Previous studies indicated that SB was significantly associated with academic performance in children and adolescents [15,16]. Nevertheless, the studies considering the various effects of different types of SB on academic achievement or physical fitness are limited, with only few presenting such relationship [17]. Meanwhile, these associations are also unclear if SB was stratified by weekdays and weekends, which will be helpful for defining high-risk factors of physical fitness and academic performance and drawing up an effective intervention target. Besides, very few studies in this research field have measured new technology-based SB (i.e., smartphone use at sitting or reclining posture). A call to action, therefore, is warranted. 

The present study aimed to explore whether SB components (screen-based, study-based, and other SB) are negatively associated with physical fitness and academic performance as well as compare their time spent on weekend to weekday days in a sample of Chinese 8–19-year-old girls and boys. 

## 2. Methods

### 2.1. Participants

A total of 1164 students aged 8–19 years from five schools (including one primary school (*n* = 215), two junior high schools (*n* = 463), and two senior high schools (*n* = 486)) participated in the study from September 2015 to May 2016 in Zhejiang, China. Finally, our study analyzed 1040 students after excluding subjects with missing variables for age or sex (*n* = 26), no informed consent (*n* = 26), or missing variables of correlates (*n* = 49) or outlier of variables (*n* = 23). All students and their parents signed the informed consent forms, and the study protocols were approved by the institutional review board of Zhejiang Financial College.

### 2.2. Questionnaire of Sedentary Behavior

The self-administered SB questionnaire was developed based on Chinese adapted scale version of the Adolescent Sedentary Activity Questionnaire [18,19] and included three domains (ten items): (a) Screen-based SB (1, 2, and 3), (b) study-based SB (4, 5, and 6), (c) other SB (7, 8, 9, and 10), see Table 1. The questionnaire was mainly completed by students but assisted by teachers or parents. The intra-class correlation coefficient of the SB questionnaire was >0.65 (for screen SB, boys: 0.80, girls: 0.84, for study SB, boys: 0.70, girls: 0.67, for other SB, boys: 0.83, girls: 0.67), which was considered as a moderate to high level, according to the recommendations of Landis et al. [20], based on an analysis of test-retest reliability. Student information, including sex, birth (age), height, weight, grade, and class, was also investigated.

### 2.3. Physical Fitness Measurements

The components of physical fitness, including vital capacity, standing long jump, 50 m run, flexibility, sit-up (for girls) or pull-up (for boys), and 800 m (for girls) or 1000 m (for boys) run tests, were performed according to the Chinese National Student Physical Fitness Standard (CNSPFS) battery [21] at the end of 2015 autumn semester. Each skill was T-scored (50 + 10 × z-score) and summed up for the final values of physical fitness. The z-score of 50 m run and 800/1000 m run have been multiplied by −1 as the lower value indicates greater performance while this is not the case in the other fitness items (higher value indicates greater performance). The measurements of all events were conducted by the registered physical education teachers. 

### 2.4. Academic Performance Measurements

Academic performance was evaluated based on four courses, including Chinese, mathematics, English, and science, which were also the official final examinations at the end of 2015 autumn semester. Each course (original test score) was T-scored (50 + 10 × z-score) and summed up for the final values of academic performance. The tests of all courses were conducted by the registered relevant academic teachers.

### 2.5. Statistical Analysis

All analyses were performed using EpiData Entry version 3.0 (The EpiData Association, Odense, Denmark) and IBM-SPSS 22.0 (IBM Inc., Armonk, NY, USA). Data were first screened for the outliers and normality of distributions. Outliers were adjusted to lessen the impact of extreme scores. Specifically, if a score or value was 3 median absolute deviations away from the median of the residuals, then that value was classified as an outlier and was truncated. Descriptive characteristics were calculated using the mean and standard deviation for continuous variables and percentages for categorical variables, unless stated otherwise. Sex differences were assessed using a T-test for continues variables of SB, physical fitness, and academic performance. Linear mixed-effects models were applied to explore associations of SB types (including screen-based SB, study-based SB, and other SB) with physical fitness and academic performance, which included fixed effects (age and SB relevant variables) and random effects (schools, grades, and classes). The interactions between the dichotomy of the week (weekdays and weekends) and SB on physical fitness or academic performance were assessed. Two points of 3 h/day and 6 h/day were applied to classify screen-based weekend SB into three categories for further analysis [22].

## 3. Results

Mean age in girls and boys was 15.02 (±2.50) and 14.76 (±2.53) years old, respectively. BMI in girls was 19.96 (±3.04) kg/m^2^, 20.76 (±3.81) kg/m^2^ in boys, 33.96 (±14.57) h/week for total SB in girls, 34.59 (±18.71) h/week in boys. Screen-based SB in boys was significantly greater than girls (including weekdays and weekends, *p* < 0.05), no significant difference was examined between boys and girls in study-based SB or other SB (*p* > 0.05), see Table 2. Boys’ scores of all components of physical fitness were greater than girls except for flexibility that was opposite (*p* < 0.001). However, girls’ academic performance was better than boys (*p* < 0.01), except for mathematics and science (*p* > 0.05). 

Associations of screen-based SB, study-based SB, and other SB with physical fitness and academic performance based on linear mixed effects model analysis are shown in Table 3. In model 1 (non-adjustment model), screen-based SB was negatively associated with physical fitness and academic performance (*p* < 0.01), study-based SB was positively associated with physical fitness and academic performance (*p* < 0.05), and other SB was positively associated with physical fitness in girls (*p* < 0.001). In model 2, after adjusting for age, the associations of study-based SB with physical fitness were not significant both in boys and girls (*p* > 0.05). In model 3, after further adjustment of school, grade, and class, the associations of screen-based SB with physical fitness were not significant in boys (*p* > 0.05). 

**Figure 1:**
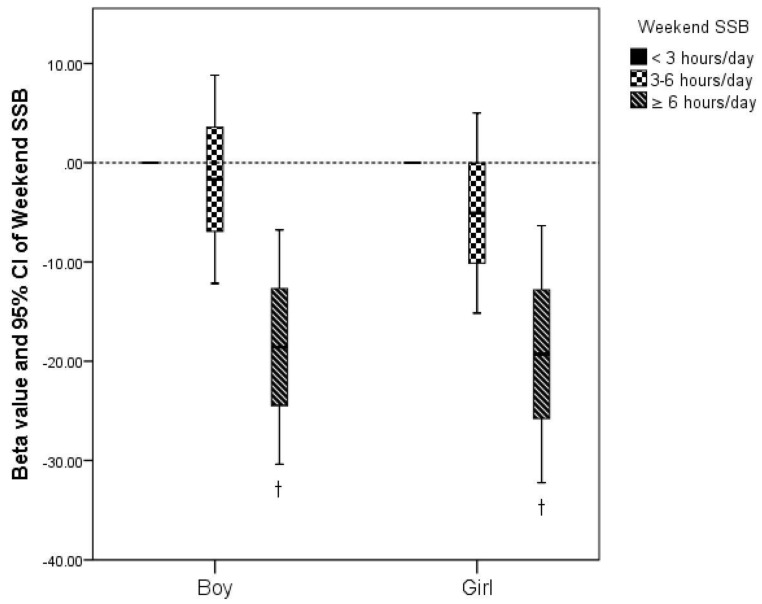
Association of screen-based sedentary behavior (SSB) on weekends with academic performance. The linear mixed effects models included weekday SSB, weekend SSB, study-based SB, other SB, variables of fixed effects model (age) and random effects model (school, grade, class). For <3 h/day, *n* = 356 (SSB = 1.43 ± 0.77 h/day, in girls), *n* = 289 (SSB = 1.42 ± 0.83 hours/day, in boys), for 3–6 h/day, *n* = 136 (SSB = 3.90 ± 0.83 h/day, in girls), *n* = 142 (SSB = 3.99 ± 0.77 h/day, in boys), for ≥6 h/day, *n* = 49 (SSB = 8.86 ± 3.13 h/day, in girls), *n* = 68 (SSB = 9.14 ± 3.63 h/day, in boys). ^†^: *p* < 0.01.

In Table 4, after adjusting for age, school, grade, class, and SB relevant variables, significant interaction of SB with weekday/weekend on academic performance in girls was observed (*p* < 0.001), while no significant associations of week day or weekend SB (including screen-based SB, study-based SB, and other SB) with physical fitness were observed (*p* > 0.05). For academic performance, however, weekend SB and screen-based weekend SB were negatively associated with academic performance in both girls and boys (*p* < 0.05). Study-based weekday SB in boys and weekday SB and study-based weekend SB in girls were positively related to academic performance (*p* < 0.05). Meanwhile, in Figure 1, screen-based weekend SB ≥ 6 h/day (screen-based weekend SB < 3 h/day as a reference group) was significantly associated with lower academic performance in both girls and boys (*p* < 0.01).

## 4. Discussion

Screen-based SB, especially on weekends, was significantly associated with poorer academic performance, and screen-based SB in girls was negatively associated with physical fitness. Study-based SB, however, was positively related with academic performance. Our results indicated that decreasing screen-based SB or replacing screen-based SB by study-based SB might be helpful for good academic performance in Chinese 8–19-years-old students.

Recent systematic reviews indicated that the relationships between SB and physical fitness were weak and inconsistent [6], although relatively strong evidence on an inverse relationship between TV viewing (≥2 h/day) and cardiorespiratory fitness, aerobic fitness, or VO_2_max were observed [23]. These inconsistencies may be due to different components of physical fitness and SB types. The evidence for the association of muscular fitness (strength) with SB was insufficient [23]. Meanwhile, significant relationships have been observed more in TV viewing than overall screen time or total SB [24]. It seems that specific relationships between SB types and physical fitness should be considered. In the present study, we observed an inverse association of screen-based SB with physical fitness in girls after adjusting for study-based SB and other SB. We found that too much time spent on “playing for recreation or leisure on a computer” played a crucial role in this relationship. This finding is significant for enriching current literature and carrying out sex-specific physical fitness intervening strategies. 

In previous studies, the association of academic performance with screen-based SB and study-based SB was consistent [7,15,16], although predicting academic achievement from overall sedentary behavior is challenging or conflicting [25,26]. In order to explore their independent effects, screen-based SB and study-based SB were simultaneously analyzed in mixed-effects regression models in our study. We found that associations of academic performance with screen-based SB and study-based SB were consistent even after controlling of SB types, physical fitness, and confounding factors in both girls and boys, while physical fitness was a significant protective factor in boys. Interestingly, only “doing homework not using computer” was related to academic performance for both sexes. Whereas, the positive relationship between “computer use for homework” and academic performance was significant only in boys. These results indicated that SB may have different effects on academic performance between girls and boys, even if time is spent on the same SB type. However, these results and their causes need to be further explored and confirmed in future cross-sectional and prospective studies.

We found that weekend time spent on screen-based SB seems much more harmful for obtaining good academic performance than time spent during weekdays. Furthermore, students with time spent on screen-based SB on weekends greater than 6 h/day had poorer academic performance (about 10%) compared with those with time spent on screen-based SB on weekends <3 h/day. To our knowledge, few studies have explored these correlations, although a study has shown that short screen time during the weekend was associated with good self-reported academic performance in Japanese children [27]. Moreover, our results indicated that the relationship between academic performance and study-based SB seems to be partially sex-specific. In our data, study-based SB on weekends was associated with academic performance (statistically significant results were shown only in mathematics) in girls but not in boys. We deduced that girls may need spending extra time (such as weekends) to study mathematics, which is a relatively difficult course for them. 

It is well known that an adequate amount of time emphasized on learning tasks is crucial for good academic performance [28]. Thus, it is possible that heavy screen-based SB displaces study-based SB, which may lead to poorer academic performance. This has been observed by many previous studies exploring the relationships between screen-based SB and academic achievement among children and adolescents [7,29,30]. For example, children who engage in high levels of TV viewing (i.e., ≥2 h/day) spend less time doing homework, learning, or reading [22]. On weekends, there is much more time that could be used to study freely. It is a critical factor determining academic performance for most students how they spend time on weekends (in front of a screen or sitting for study). 

Some limitations should be noted in our study. First, a self-report questionnaire was applied to measure time spent on activities of SB, which may cause measurement bias, although general objective tools (e.g., ActiGraph Link GT9X) could not provide the information about types or context of SB [31]. Second, various scales were used to measure students’ academic performance in different schools and grades. To minimize the potential bias, the standardized data of SB and physical fitness were used as well as handled variables of school, grade, and class as the independent variables in random-effects model in the final analysis. The mixed-effects model was considered as a great statistics tool to deal with data clustering problem. Third, however, our results based on a cross-sectional study could not infer their causal relationships. 

## 5. Conclusions

Heavy screen-based SB, especially on weekends, was an important risk factor for poorer academic performance, whereas the study-based SB was positively associated with academic performance in Chinese 8–19-years-old students. Future strategies aiming to promote children’s academic performance should focus on limiting screen-based SB. The prospective associations of the components of SB with academic performance and physical fitness should be further explored in future studies.

## Figures and Tables

**Table 1:** Sedentary behavior questionnaire for Chinese children and adolescents.

No.	Items	Do You Participate in This Activity?	How Much Time Do You Participate in This Activity?	
			Monday–Friday	Saturday–Sunday
1	Television or video viewing	☐Yes ☐No	hours minutes	hours minutes
2	Playing for recreation or leisure on a Cell phone or tablet	☐Yes ☐No	hours minutes	hours minutes
3	Playing for recreation or leisure on a computer	☐Yes ☐No	hours minutes	hours minutes
4	Computer use for homework	☐Yes ☐No	hours minutes	hours minutes
5	Doing homework not using computer	☐Yes ☐No	hours minutes	hours minutes
6	Attending extracurricular classes	☐Yes ☐No	hours minutes	hours minutes
7	Reading for fun or interest	☐Yes ☐No	hours minutes	hours minutes
8	Transportation by car or bus or train	☐Yes ☐No	hours minutes	hours minutes
9	Talking on the phone or sit around	☐Yes ☐No	hours minutes	hours minutes
10	Others such as practicing musical instruments, calligraphy/painting, making crafts, listening to music, etc.	☐Yes ☐No	hours minutes	hours minutes

**Table 2:** Characteristics of Chinese students aged 8–19 years.

Variables	Girls M (SD)	Boys M (SD)	*p* Value
*n*	541 (52.02)	499 (47.98)	
Age, years	15.02 (2.50)	14.76 (2.53)	0.089
Height, cm	159.76 (6.08)	167.96 (9.60)	<0.001
Weight, kg	51.27 (9.29)	58.91 (13.85)	<0.001
BMI, kg/m^2^	19.96 (3.04)	20.76 (3.81)	<0.001
Sedentary behavior, hours/week			
Screen-based SB	6.56 (5.99)	7.96 (8.66)	0.002
Weekday	1.10 (2.13)	1.55 (3.75)	0.016
Weekend	5.45 (5.01)	6.40 (6.06)	0.006
Study-based SB	17.62 (10.89)	16.54 (12.15)	0.130
Weekday	11.54 (8.69)	10.86 (9.67)	0.233
Weekend	6.09 (4.43)	5.68 (4.46)	0.140
Other SB	9.78 (7.48)	10.09 (10.16)	0.569
Weekday	4.42 (4.20)	5.03 (7.28)	0.093
Weekend	5.37 (4.42)	5.07 (4.95)	0.100
Total SB	33.96 (14.57)	34.59 (18.71)	0.541
Physical fitness			
Vital capacity, ml	2743.17 (581.78)	3555.39 (1034.32)	<0.001
Standing long jump, cm	174.44 (21.49)	213.79 (34.98)	<0.001
50 m run, seconds	8.83 (0.70)	7.83 (0.97)	<0.001
Flexibility, cm	14.52 (7.03)	9.13 (8.35)	<0.001
Sit-up, times	39.15 (10.43)	/	/
Pull-up, times	/	4.60 (5.58)	/
800 m, seconds	228.90 (21.92)	/	/
1000 m run, seconds	/	253.87 (40.01)	/
Academic Performance			
Chinese, scores	80.45 (12.28)	75.77 (13.54)	<0.001
Mathematics, scores	70.71 (22.63)	70.67 (24.68)	0.984
English, scores	80.62 (18.48)	69.88 (21.82)	<0.001
Science, scores	75.70 (31.70)	76.57 (32.28)	0.787

M (SD), mean (standard deviation); BMI, body mass index; SB, sedentary behavior.

**Table 3:** Associations of screen-based SB, study-based SB, and other SB with physical fitness and academic performance.

Models	Physical Fitness		Academic Performance	
	Girls β (SE)	Boys β (SE)	Girls β (SE)	Boys β (SE)
Model 1				
Screen-based SB	−0.97 ^‡^ (0.22)	−0.66 ^†^ (0.25)	−1.57 ^‡^ (0.32)	−1.04 ^‡^ (0.21)
Study-based SB	0.33 ^*^ (0.13)	0.93 ^‡^ (0.19)	0.55 ^*^ (0.23)	0.68 ^†^ (0.24)
Other SB	0.66 ^‡^ (0.18)	0.39 (0.23)	0.32 (0.32)	−0.09 (0.21)
Model 2				
Screen-based SB	−0.61 ^†^ (0.21)	−0.38 ^*^ (0.17)	−1.50 ^‡^ (0.32)	−0.89 ^‡^ (0.21)
Study-based SB	0.11 (0.12)	−0.02 (0.14)	0.58 ^*^ (0.23)	0.64 ^†^ (0.23)
Other SB	0.47 ^†^ (0.16)	0.15 (0.16)	0.25 (0.32)	−0.08 (0.21)
Model 3				
Screen-based SB	−0.46 ^*^ (0.20)	−0.22 (0.17)	−1.19 ^‡^ (0.28)	−0.82 ^‡^ (0.19)
Study-based SB	0.12 (0.12)	−0.05 (0.14)	0.67 ^†^ (0.20)	0.77 ^‡^ (0.22)
Other SB	0.38 ^*^ (0.16)	0.21 (0.15)	0.18 (0.28)	−0.09 (0.20)

β (SE), beta (standard error); SB, sedentary behavior. Model 1: Non-adjustment model (independent variables included screen-based SB, study-based SB, and other SB). Model 2: Model 1 + age. Model 3: Model 2 + school, grade, and class. *: *p* < 0.05; ^†^: *p* < 0.01; ^‡^: *p* < 0.001.

**Table 4:** Associations of SB spent during weekdays and weekends with physical fitness and academic performance.

Models ^#^	Physical Fitness		Academic Performance	
	Girls β (SE)	Boys β (SE)	Girls β (SE)	Boys β (SE)
Model 1				
Total SB	0.25 (0.25)	−0.14 (0.25)	1.63 ^‡^ (0.48)	0.36 (0.37)
Weekday/weekend	2.24 (3.45)	−2.46 (3.62)	18.21 ^†^ (6.18)	5.71 (4.87)
Weekday/weekend × Total SB	−0.12 (0.17)	0.13 (0.16)	−1.11 ^‡^ (0.33)	−0.31(0.22)
Model 2				
Weekday SB	0.20 (0.12)	0.01 (0.13)	0.70 ^†^ (0.21)	0.23 (0.20)
Weekend SB	−0.01 (0.16)	−0.03 (0.19)	−0.83 ^†^ (0.27)	−0.56^*^(0.26)
Model 3				
Screen-based SB				
Weekday	−0.85 (0.56)	0.10 (0.45)	−0.23 (0.78)	−0.60(0.51)
Weekend	−0.36 (0.25)	−0.37 (0.29)	−1.43 ^‡^ (0.36)	−0.99^†^(0.36)
Study-based SB				
Weekday	0.16 (0.15)	−0.07 (0.19)	0.50 (0.26)	0.99 ^†^(0.32)
Weekend	0.02 (0.31)	0.06 (0.36)	1.23 ^*^ (0.60)	0.33 (0.57)
Other SB				
Weekday	0.30 (0.32)	0.11 (0.22)	0.53 (0.55)	−0.16(0.26)
Weekend	0.44 (0.30)	0.38 (0.32)	−0.10 (0.62)	0.17 (0.49)

β (SE), beta (standard error); SB, sedentary behavior. Model 1, model 2, and model 3 adjusted for variables of fixed effects model (age) and random effects model (school, grade, class). *: *p* < 0.05; ^†^: *p* < 0.01; ^‡^: *p* < 0.001.

## List of Abbreviations

SB	Sedentary Behavior
METs	Metabolic Equivalent of Tasks
MVPA	Moderate or Vigorous Physical Activity
TV	Television
BMI	Body Mass Index
